# *In Vitro* Synergy Testing of Eravacycline in Combination with Clarithromycin and Rifabutin against Mycobacterium abscessus Complex

**DOI:** 10.1128/spectrum.00045-21

**Published:** 2021-06-16

**Authors:** Ka Lip Chew, Sophie Octavia, Siang Fei Yeoh, Jeanette W. P. Teo

**Affiliations:** a Department of Laboratory Medicine, National University Hospital, Singapore; b Environmental Health Institute, National Environment Agency, Singapore; c Department of Pharmacy, National University Hospital, Singapore; University of Manitoba

**Keywords:** checkerboard broth microdilution, clofazimine, eravacycline, *Mycobacterium abscessus* complex, rifabutin, synergy

## LETTER

The Mycobacterium abscessus complex (MabsC) is a group of multidrug-resistant nontuberculous mycobacteria (NTM) which may cause pulmonary and extrapulmonary infections (1). It consists of three subspecies: Mycobacterium abscessus, Mycobacterium massiliense, and Mycobacterium bolletii. Treatment outcomes are often poor, with clarithromycin susceptibility (largely mediated by the *erm41* gene) being one of the key factors influencing treatment outcome (2, 3). Clarithromycin is usually recommended as the key drug in treatment regimens and is used in combination with other antibiotics based on antimicrobial susceptibility testing (4). However, the organism is often resistant to various antibiotics (5), and there is interest in exploring new antimicrobials with potential antimycobacterial activity against MabsC.

*In vitro* activity of eravacycline has been demonstrated against MabsC (5, 6). Eravacycline is a fluorocycline which is an analogue of tetracyclines. Despite intrinsic resistance to tetracyclines with little inhibition in the usual tested ranges (MIC_50_ of >16 mg/liter) (5), eravacycline MICs occur between 0.015 and 2 mg/liter. Another antimicrobial of interest is rifabutin. In addition to its having lower MICs against MabsC than other rifamycins, synergy has been demonstrated with clarithromycin, mediated by inhibition of expression of the *whiB7* regulator of *erm*(41) (7, 8). In this study, we explored synergy of eravacycline in combination with rifabutin and clarithromycin.

Checkerboard testing was performed between eravacycline against clarithromycin, rifabutin, clofazimine, and bedaquiline, using cation-adjusted Mueller-Hinton broth (BD Diagnostics, Franklin Lakes, NJ, USA). Antibiotic powders were purchased from Medchem Express (Monmouth Junction, NJ, USA). Testing was performed in 96-well microtiter plates using a final volume of 100 μl, with a final bacterial inoculum size of 5 × 10^4^ CFU. Plates were incubated under ambient conditions at 30°C. The eravacycline-rifabutin combination was incubated to day 5, while the eravacycline-clarithromycin combination was incubated to day 14, as recommended by CLSI for testing inducible clarithromycin resistance. The plates were read visually, with MICs read at 100% inhibition (no visible bacterial growth). The fractional inhibitory concentration index (FICI) was calculated; a FICI of ≤0.5 was defined as synergistic, a FICI of 0.5 of 4 as indifferent, and a FICI of >4 as antagonistic (9).

Twenty-three clinical isolates (10 Mycobacterium abscessus, 10 Mycobacterium massiliense, and three Mycobacterium bolletii isolates) were chosen from a larger collection of isolates for which whole-genome sequencing was performed on the Illumina Hiseq sequencing platform (Illumina, Inc., San Diego, CA, USA). The average sequencing depth was 150×. Species identification was determined by phylogenetic analyses, and multilocus sequence typing (MLST) was performed using the scheme for Mycobacterium abscessus (https://github.com/phac-nml/mab_mabscessus). Isolates with different sequence types were selected to represent genomically diverse isolates.

The results are summarized in Table 1. Eravacycline was neither synergistic nor antagonistic in combination with rifabutin and clarithromycin. Only one isolate had an FICI of ≤0.5 for the eravacycline-rifabutin combination. Despite synergy not being demonstrated on the checkerboard assay, there is still potential for inclusion of eravacycline in treatment regimens for MabsC infections. No antagonism was seen with the key antimicrobial clarithromycin or rifabutin as a potential antibiotic repurposed to treat MabsC. Additional data are required to further explore this with animal models or clinical data.

**TABLE 1:** MIC and FICI results for eravacycline tested in combination with rifabutin and clarithromcyin^*a*^

Species	Isolate	ST	*erm*(41) sequevar	MIC (mg/liter)				FICI	MIC (mg/liter)				FICI
				Alone		In combination (ERV + RFB)			Alone		In combination (ERV + CLR)		
				ERV	RFB	ERV	RFB		ERV	CLR	ERV	CLR	
M. abscessus	RGM2	28	WT	0.06	1	0.03	1	1.5	0.06	32	0.03	32	1.5
	RGM32	62	T28C	0.06	2	0.06	1	1.5	0.03	0.5	0.06	0.5	3
	RGM34	101	WT	0.12	1	0.12	1	2	0.06	32	0.03	32	1.5
	RGM44	147	T28C	0.12	2	0.12	1	1.5	0.12	0.25	0.12	0.25	2
	RGM60	31	WT	0.12	4	0.06	1	0.75	0.12	16	0.12	16	2
	RGM66	34	WT	0.06	2	0.12	1	2.5	0.06	32	0.12	32	3
	RGM72	5	WT	0.12	2	0.12	1	1.5	0.12	32	0.12	32	2
	RGM121	40	T28C	0.12	2	0.03	1	0.75	0.06	0.25	0.03	0.25	1.5
	RGM129	245	T28C	0.12	4	0.06	1	0.75	0.12	0.25	0.03	0.25	1.25
	RGM218	23	T28C	0.06	4	0.06	1	1.25	0.12	0.5	0.12	0.5	2

*M. bolletii*	RGM7	6	WT	0.12	4	0.12	1	1.25	0.12	0.25	0.03	0.25	1.25
	RGM217	54	WT	0.25	4	0.25	1	1.25	0.03	0.25	0.03	0.25	2
	RGM238	10	WT	0.12	2	0.06	1	1	0.12	0.25	0.03	0.25	1.25

*M. massiliense*	RGM19	76	`NA	0.25	4	0.06	1	0.5	0.06	0.25	0.03	0.25	1.5
	RGM51	29	`NA	0.12	2	0.03	1	0.75	0.12	0.25	0.03	0.25	1.25
	RGM68	186	`NA	0.06	4	0.06	1	1.25	0.12	0.25	0.03	0.25	1.25
	RGM112	12	`NA	0.12	2	0.06	1	1	0.12	0.25	0.03	0.25	1.25
	RGM122	62	`NA	0.25	4	0.12	1	0.75	0.12	0.25	0.03	0.25	1.25
	RGM123	32	`NA	0.06	4	0.06	1	1.25	0.06	0.25	0.03	0.25	1.5
	RGM139	4	`NA	0.5	8	0.5	1	1.125	0.25	0.25	0.06	0.25	1.25
	RGM163	7	`NA	0.06	8	0.06	1	1.125	0.12	0.25	0.06	0.25	1.5
	RGM167	5	`NA	0.12	4	0.06	1	0.75	0.06	0.25	0.06	0.25	2
	RGM233	100	`NA	0.12	2	0.03	1	0.75	0.03	0.25	0.03	0.25	2

a ST, Sequence type; ERV, eravacycline; RFB, rifabutin; CLR, clarithromycin, FICI, fractional-inhibitory-concentration index; WT, wild type; NA, not applicable due to truncated *erm41*.
